# APP intracellular domain derived from amyloidogenic β- and γ-secretase cleavage regulates neprilysin expression

**DOI:** 10.3389/fnagi.2015.00077

**Published:** 2015-05-19

**Authors:** Marcus O. W. Grimm, Janine Mett, Christoph P. Stahlmann, Sven Grösgen, Viola J. Haupenthal, Tamara Blümel, Benjamin Hundsdörfer, Valerie C. Zimmer, Nadine T. Mylonas, Heikki Tanila, Ulrike Müller, Heike S. Grimm, Tobias Hartmann

**Affiliations:** ^1^Department of Experimental Neurology, Saarland UniversityHomburg, Germany; ^2^Department of Neurodegeneration and Neurobiology, Saarland UniversityHomburg, Germany; ^3^Deutsches Institut für DemenzPrävention, Saarland UniversityHomburg, Germany; ^4^Department of Neurobiology, A.I. Virtanen Institute, University of Eastern FinlandKuopio, Finland; ^5^Department of Neurology, Kuopio University HospitalKuopio, Finland; ^6^Department of Functional Genomics, Institute for Pharmacy and Molecular Biotechnology, Heidelberg UniversityHeidelberg, Germany

**Keywords:** neprilysin, Alzheimer's disease, APP intracellular domain, AICD, gene regulation, Aβ degradation

## Abstract

Alzheimer's disease (AD) is characterized by an accumulation of Amyloid-β (Aβ), released by sequential proteolytic processing of the amyloid precursor protein (APP) by β - and γ-secretase. Aβ peptides can aggregate, leading to toxic Aβ oligomers and amyloid plaque formation. Aβ accumulation is not only dependent on *de novo* synthesis but also on Aβ degradation. Neprilysin (NEP) is one of the major enzymes involved in Aβ degradation. Here we investigate the molecular mechanism of NEP regulation, which is up to now controversially discussed to be affected by APP processing itself. We found that NEP expression is highly dependent on the APP intracellular domain (AICD), released by APP processing. Mouse embryonic fibroblasts devoid of APP processing, either by the lack of the catalytically active subunit of the γ-secretase complex [presenilin (PS) 1/2] or by the lack of APP and the APP-like protein 2 (APLP2), showed a decreased NEP expression, activity and protein level. Similar results were obtained by utilizing cells lacking a functional AICD domain (APPΔCT15) or expressing mutations in the genes encoding for PS1. AICD supplementation or retransfection with an AICD encoding plasmid could rescue the down-regulation of NEP further strengthening the link between AICD and transcriptional NEP regulation, in which Fe65 acts as an important adaptor protein. Especially AICD generated by the amyloidogenic pathway seems to be more involved in the regulation of NEP expression. In line, analysis of NEP gene expression *in vivo* in six transgenic AD mouse models (APP and APLP2 single knock-outs, APP/APLP2 double knock-out, APP-swedish, APP-swedish/PS1Δexon9, and APPΔCT15) confirmed the results obtained in cell culture. In summary, in the present study we clearly demonstrate an AICD-dependent regulation of the Aβ-degrading enzyme NEP *in vitro* and *in vivo* and elucidate the underlying mechanisms that might be beneficial to develop new therapeutic strategies for the treatment of AD.

## Introduction

Alzheimer's disease (AD) is a progressive, irreversible neurodegenerative disease of the central nervous system and the most common cause of dementia in the aged population. One of the focal histopathological hallmarks of AD are extracellular senile plaques, mainly consisting of aggregated amyloid-β (Aβ) peptides (Glenner and Wong, 1984; Masters et al., 1985). Aβ peptides are derived from sequential proteolytic processing of the amyloid precursor protein (APP), member of a conserved protein family also including the APP-like proteins 1 and 2 (APLP1 and APLP2) (Wasco et al., 1992, 1993), by β - and γ-secretase (Selkoe, 2001; Sisodia and St George-Hyslop, 2002). Initial β-secretase shedding of APP releases soluble β-secreted APP fragments (sAPPβ) and generates a membrane-tethered C-terminal fragment of APP, called β-CTF, which is further cleaved by γ-secretase, generating Aβ peptides of different lengths (e.g., Aβ37, Aβ40, Aβ42, Aβ46, Aβ49) (Wang et al., 1996; Zhao et al., 2007; Schieb et al., 2011). The γ-secretase activity involves a heterotetrameric protein complex (Edbauer et al., 2003; Kimberly et al., 2003; Takasugi et al., 2003) with the presenilins, presenilin 1 (PS1) and 2 (PS2), as catalytically active subunits (Herreman et al., 2000; Zhang et al., 2000; Ahn et al., 2010). Beside β-secretase cleavage of APP, APP can be first cleaved by α-secretases in a non-amyloidogenic pathway, preventing the generation of Aβ peptides. The α-secretases process APP within the Aβ domain, generating α-secreted APP (sAPPα) and the C-terminal fragment α-CTF, further cleaved by γ-secretase to generate p3 (Esch et al., 1990; Haass et al., 1993; Lichtenthaler, 2011). Processing of α-CTF and β-CTF fragments by the γ-secretase complex also results in the release of the APP intracellular domain (AICD) to the cytosol which is discussed to be involved in gene transcription (Passer et al., 2000; Gao and Pimplikar, 2001; Cao and Sudhof, 2004; von Rotz et al., 2004; Pardossi-Piquard and Checler, 2012). Multiple-site cleavages executed by the γ-secretase complex generate accordingly AICDs of various lengths including C50, C53, C57, and C59 (Gu et al., 2001; Pinnix et al., 2001; Sastre et al., 2001; Yu et al., 2001; Sato et al., 2003; Zhang et al., 2012; Pinnix et al., 2013). However, all of the endogenous AICD isoforms are rarely detected, because of their rapid degradation in the cytosol by insulin degrading enzyme (IDE) (Edbauer et al., 2002; Farris et al., 2003; Miller et al., 2003), the proteasome (Nunan et al., 2003) and Cathepsin B (Vingtdeux et al., 2007; Asai et al., 2011). Rapid binding of AICD peptides to adaptor proteins like Fe65 might stabilize released AICD peptides (Kimberly et al., 2001; Kinoshita et al., 2002), enabling AICD to translocate to the nucleus and to form a trimeric protein complex with the histone acetyltransferase Tip60 (AFT-complex) (Cao and Sudhof, 2001; von Rotz et al., 2004; Grimm et al., 2013). Several potential target genes of AICD nuclear signaling have been identified (Grimm et al., 2013), including e.g. enzymes of different lipid pathways (Grimm et al., 2011a,c, 2012b), glycogen synthase kinase-3β (Kim et al., 2003), lipoprotein receptor LRP1 (Liu et al., 2007), and mitochondrial master transcriptional coactivator PGC1α (Robinson et al., 2014). Interestingly, AICD is also discussed to regulate transcription of APP and the β-secretase BACE1 as well as the transcription of neprilysin (NEP) (von Rotz et al., 2004; Pardossi-Piquard et al., 2005), beside IDE (Farris et al., 2003; Hersh, 2006) one of the most prominent Aβ-degrading enzymes (Iwata et al., 2000, 2001; Hersh and Rodgers, 2008). For AICD mediated activation of NEP gene expression an epigenetical mechanism involving the competitive replacement of histone deacetylases (HDAC) at the NEP regulatory site has been demonstrated (Belyaev et al., 2009; Nalivaeva et al., 2012). However, the impact of AICD in transcriptional regulation of NEP is still controversially discussed (Grimm et al., 2013) (summarized in Table 1). Summing up, the results in Table 1 clearly show how controversially the impact of AICD on NEP regulation is discussed in literature. The aim of this study was to comprehensively elucidate a possible regulation of NEP expression by AICD. Besides addressing this aim with known and published systems we generated broad pharmacological and genetic approaches to unambiguously elucidate the role of AICD in NEP regulation. Moreover, it was important for us to confirm our findings *in vivo* in several transgenic mouse models. Additionally, we found further evidences supporting the underlying mechanisms of AICD mediated NEP regulation. These mechanisms might also be involved in the regulation of other transcriptional targets discussed in literature, which are mentioned above. In respect to AD it is important to notice that NEP has been shown to be reduced in brain areas early affected in AD and characterized by extensive plaque load (Akiyama et al., 2001; Yasojima et al., 2001; Carpentier et al., 2002; Russo et al., 2005; Wang et al., 2005; Miners et al., 2006) indicating the significance of understanding the regulation of this Aβ degrading enzyme.

**Table 1:** **Summary of studies elucidating the impact of γ-secretase and members of the APP protein family on NEP resulting in inhomogeneous outcomes (↓, decreased; -, no effect; Δ, genetic deletion)**.

**Used celllines/ Mousemodels**	**NEP expression**	**NEP level**	**NEP activity**	**Rescue?**
MEF ΔPS1ΔPS2	↓ (Pardossi-Piquard et al., 2005)	↓ (Pardossi-Piquard et al., 2005)	↓ (Pardossi-Piquard et al., 2005)	By expression of PS1, PS2, PS1+PS2 and AICD50,AICD59; higher effects by expression of AICD50/59+ Fe65+ Tip60 (Pardossi-Piquard et al., 2005);
	− (Huysseune et al., 2009)	− (Hébert et al., 2006)		Not by expression of PS1 (Chen and Selkoe, 2007)
		↓ (Chen and Selkoe, 2007)		
BD8 ΔPS1ΔPS2		↓ (Pardossi-Piquard et al., 2005)	↓ (Pardossi-Piquard et al., 2005)	By expression of AICD50 and AICD59 (Pardossi-Piquard et al., 2005);
		− (Chen and Selkoe, 2007)		Not by expression of PS1, PS2 or AICD60+ Fe65+ Tip60 (Chen and Selkoe, 2007)
ΔPS1ΔPS2 (conditional) mouse brain		↓ (Pardossi-Piquard et al., 2005)	↓ (Pardossi-Piquard et al., 2005)	
ΔPS1 mouse embryo brain (E14, 5)		− (Hébert et al., 2006)		
ΔPS1 mouse brain			− (Pardossi-Piquard et al., 2005)	
MEF ΔAph1a		− (Hébert et al., 2006)		
ΔAph1a whole mouse embryo (E9, 5)		− (Hébert et al., 2006)		
γ-secretase inhibition in MEF WT		− with DAPT and γ-secretase inhibitor X (Hébert et al., 2006)	↓ with several γ-secretase inhibitors (Pardossi-Piquard et al., 2005)	
		− with DAPT (Chen and Selkoe, 2007)		
γ-secretase inhibition in TSM1 neurons			↓ with DFK167 (Pardossi-Piquard et al., 2005)	
γ-secretase inhibition in primary cultured neurons			↓ with DFK167 (Pardossi-Piquard et al., 2005)	
γ-secretase inhibition in Hela WT, cos7 WT and N2a WT		− with DAPT and γ-secretase inhibitor X (Hébert et al., 2006)		
γ-secretase inhibition in BD8 WT and HEK293T WT		− with Compound E (Chen and Selkoe, 2007)		
γ-secretase inhibition in NB7 cells and SK-N-SH cells	↓ with DAPT (Xu et al., 2011)			
MEFΔAPPΔAPLP2	↓ (Pardossi-Piquard et al., 2005)	↓ (Pardossi-Piquard et al., 2005)	↓ (Pardossi-Piquard et al., 2005)	By expression of ALID1 or ALID2 or AICD50 (Pardossi-Piquard et al., 2005)
		− (Hébert et al., 2006)		
		− (Huysseune et al., 2009)		
MEFΔAPP	↓ (Pardossi-Piquard et al., 2005)	↓ (Pardossi-Piquard et al., 2005)	↓ (Pardossi-Piquard et al., 2005)	By expression of APP (Pardossi-Piquard et al., 2005)
	− (Huysseune et al., 2009)	− (Huysseune et al., 2009)		
MEFΔAPLP2	↓ (Pardossi-Piquard et al., 2005)	↓ (Pardossi-Piquard et al., 2005)	↓ (Pardossi-Piquard et al., 2005)	By expression of APLP2 (Pardossi-Piquard et al., 2005)
APP knockdown in NB7 cells	↓ (Belyaev et al., 2009)			
	↓ (Xu et al., 2011)			
APP knockdown in SK-N-SH cells	↓ (Xu et al., 2011)			
ΔAPPΔAPLP2 mouse brain			↓ (Pardossi-Piquard et al., 2005)	
ΔAPPΔAPLP2 embryonic brain (E15, 5)	− (Hébert et al., 2006)	− (Hébert et al., 2006)		
ΔAPPΔAPLP1 mouse brain			↓ (Pardossi-Piquard et al., 2005)	
ΔAPP mouse brain		− (Chen and Selkoe, 2007)	↓ (Pardossi-Piquard et al., 2005)	
			− (Chen and Selkoe, 2007)	
ΔAPLP2 mouse brain		− (Chen and Selkoe, 2007)	− (Chen and Selkoe, 2007)	

## Materials and methods

### Chemicals

Chemicals and cell culture materials were purchased from Sigma (Taufkirchen, Germany) if not stated otherwise.

### Cell culture

#### Cultivation

Human neuroblastoma SH-SY5Y wildtype (wt) cells were cultivated in Dulbecco's modified Eagle's medium (DMEM) containing 10% fetal calf serum (FCS) (PAN Biotech, Aidenbach, Germany) and 0.1 mM non-essential amino acid solution (MEM). For SH-SY5Y cells stably overexpressing APP/APPswe and α-/β-CTF, culture medium was supplemented with 400 μg/ml hygromycin B (PAN Biotech, Aidenbach, Germany) or 300 μg/ml zeocin (Invitrogen, Karlsruhe, Germany), respectively. SH-SY5Y Fe65 knock-down, IDE knock-down and the corresponding mock-transfected control cells were also cultivated in 10% FCS/DMEM supplemented with hygromycin B.

Wildtype mouse embryonic fibroblasts (MEF wt), PS1/2 deficient MEF (MEF PS1/2^−/−^) (Herreman et al., 2000), APP/APLP2 deficient MEF (MEF APP/APLP2^−/−^) (Heber et al., 2000) and MEF expressing an APP construct that lacks the last 15 amino acids (aa) from the C-terminus (MEF APPΔCT15)(Ring et al., 2007) were cultivated in 10% FCS/DMEM. For MEF PS1/2^−/−^ retransfected with PS1 wt or PS1 carrying the familial AD (FAD) T354I mutation (Grimm et al., 2005), cell culture medium was supplemented with 300 μg/ml zeocin.

#### Incubations

Cells were cultured until attaining confluence. 16 h prior to incubation FCS content of the culture medium was reduced to 1%. Then cells were treated with 5 μM α-secretase inhibitor GM6001 (Calbiochem, Darmstadt, Germany), 500 nM β-secretase inhibitor IV (Calbiochem, Darmstadt, Germany), 2 μM γ-secretase inhibitor X (Calbiochem, Darmstadt, Germany) or 10 μM human insulin for 24 h with a medium change after 12 h in 1% FCS/DMEM; control cells were treated with the corresponding solvent.

To determine the effects of AICD peptide, MEF APPΔCT15 cells were treated with synthetic AICD peptide (sequence in 1-letter code: KMQQNGYENPTYKFFEQMQN) (Genscript Corporation, Piscatway, USA). For long term AICD incubation 2 μM AICD was incubated on cells over a period of 9 days with medium change every 12 h in 10% FCS/DMEM; short time incubation (12 h) was performed by using 2,5 μM AICD and the protein delivery reagent Saint PhD (Synvolux Therapeutics, Groningen, The Netherlands) according to manufacturer's protocol. For AICD uptake analysis FITC-tagged AICD peptides (Genscript Corporation, Piscatway, USA) were used for incubations. Incubation of MEF with human Aβ40/42 peptides in a physiological ratio was performed in analogy to long term AICD incubation. The final concentrations of Aβ40 and Aβ42 in cell culture medium were 40 and 4 ng/ml, respectively.

#### Transfections

All transfections were performed by using Lipofectamine 2000 and Opti-MEM (Invitrogen, Karlsruhe, Germany) according to manufacturer's instructions. 48 h after transfection, cells were harvested in the case of transient transfections or selection for stable clones was started.

#### Knock-down experiments

SH-SY5Y Fe65 knock-down cells were described earlier (Grimm et al., 2011a). SH-SY5Y IDE knock-down and the corresponding control cells were generated by transfection with SureSilencingTM–Insulin degrading enzyme shRNA plasmid and control vector (SABioscience, Frederick, MD, USA) according to manufacturer's protocol followed by selection for stable clones with hygromycin B.

### Murine brain material

Murine APP^−/−^, APLP2^−/−^, APP/APLP2^−/−^, and APPΔCT15 brain material was obtained from U. Müller (Heidelberg, Germany), APPswe/PS1Δexon9 and APPswe mouse brains from H. Tanila (Kuopio, Finland).

### Quantitative real-time PCR (RT-PCR)

TRIzolReagent (Invitrogen, Karlsruhe, Germany) was used for extraction of total RNA from cells and tissue. 2 μg RNA was reverse-transcribed using the High-Capacity cDNA Reverse Transcription Kit (Life Technologies, Darmstadt, Germany) according to manufacturers' protocols. Quantitative real-time PCR was performed by application of Fast SYBR Green Master Mix (Applied Biosystems, Darmstadt, Germany) and Piko Real Real-Time PCR System (Thermo Scientific, Waltham, USA). After normalization to β-actin, changes in gene expression were calculated with the 2-(ΔΔCt) method (Livak and Schmittgen, 2001). The following primer sequences were used (Eurofins MWG Operon, Eberberg, Germany): murine: β-actin 5′-CCT AGG CAC CAG GGT GTG AT-3′ and 5′-TCT CCA TGT CGT CCC AGT TG-3′; insulin degrading enzyme 5′-GCT ACG TGC AGA AGG ACC TC-3′ and 5′-TGG ACG TAT AGC CTC GTG GT-3′; neprilysin 5′-TGA ACT TTG CCC AGG TGT G-3′ and 5′-GCA AAG TCC CAA TGA TCC TG-3′; human: β-actin 5′-CTT CCT GGG CAT GGA GTC-3′ and 5′-AGC ACT GTG TTG GCG TAC AG-3′; Fe65 5′-TTT GGA AGG ATG AAC CCA GT-3′ and 5′-AAG CTT CTC CTC CTC TTG GG-3′; insulin degrading enzyme 5′- TGC CCT AGA CAG GTT TGC AC-3′ and 5′-CTC CAG GCA TCA TTC ATC ACA T-3′; neprilysin 5′-GAT CAG CCT CTC GGT CCT TG-3′ and 5′-TGT TTT GGA TCA GTC GAG CAG-3′.

### Preparation of cell lysates and collection of conditioned media

Cells were washed with phosphate-buffered saline (PBS), scraped off and lysed chemically for 1 h with different lysis buffers depending on further usage. Conditioned media were collected and centrifuged for 5 min at 16.000 × g, the supernatants were applied for determination of Aβ, sAPPα, and sAPPβ level by western blot analysis.

### Membrane preparation of eukaryotic cells

After incubation of pelleted cells in hypotonic buffer (10 mM Tris pH 7.6, 1 mM EDTA, 1 mM EGTA) for 15 min, cell suspension was passed through 0.6 mm-needles (BD, Franklin Lakes, NJ, USA). Samples were centrifuged for 5 min at 300 × g, supernatant was collected and centrifuged for further 30 min at 12.000 × g. Pelleted membranes were resuspended in lysis buffer (150 mM NaCl, 50 mM Tris/HCl pH 7.4, 2 mM EDTA, 0.1% NP-40, 0.1% Triton-X 100) and further used for NEP western blot analysis.

### Protein determination

Protein determination was carried out using bicinchoninic acid according to Smith et al. (1985) as described in detail earlier (Rothhaar et al., 2012). Samples were adjusted to equal protein amount prior to further usage in experiments.

### Western blot analysis

Cells were lysed in lysis buffer (150 mM NaCl, 50 mM Tris/HCl pH 7.4, 2 mM EDTA, 0.1% NP-40, 0.1% Triton-X 100) containing protease inhibitor cocktail (Roche Diagnostics, Mannheim, Germany), adjusted to equal protein amount and loaded on 10–20% Tricine gels (Anamed Elektrophorese, Groß-Bieberau, Germany). Proteins were transferred onto nitrocellulose membranes (Whatman, Dassel, Germany). For Western blot analysis the following primary antibodies were used: anti-sAPPβ MBS492139 (MyBioSource, SanDiego, USA), anti-NEP ab951 (Abcam, Cambridge, UK) and W02-antibody (Millipore, Billerica, USA) for detection of sAPPα and immunoprecipitated Aβ as described earlier (Grimm et al., 2011b, 2015). Anti-rabbit W401 (Promega, Mannheim, Germany) and anti-mouse P0260 (Dako, Hamburg, Germany) were used as secondary antibodies. Proteins were detected by ECL-method (Perkin Elmer, Rodgau-Jügesheim, Germany), densitometric quantification was performed with Image Gauge V3.45 software.

### Analysis of AICD uptake

For AICD peptide uptake analysis in cell lysates MEF APPΔCT15 were incubated with FITC-AICD as decribed above. Under light exclusion cells were washed 5 times with PBS to remove attached FITC-AICD peptides and lysed in lysis buffer (150 mM NaCl, 50 mM Tris/HCl pH 7.4, 2 mM EDTA, 0.1% NP-40, 0.1% Triton-X 100). Lysates were adjusted to equal protein content and dispensed on black 96-well plates (Corning, Lowell, MA, USA). Fluorescence signal of FITC-AICD was determined at an excitation wavelength of 495 ± 10 nm and an emission wavelength of 521 ± 10 nm using an Infinite M1000Pro Fluorometer (Tecan, Crailsheim, Germany).

### Cell viability measurement

Cell viability after short term incubation with AICD peptides was determined by measuring lactate dehydrogenase (LDH) activity using the Cytotoxicity Detection KitPLUS (Roche Diagnostics, Mannheim, Germany) according to manufacturer's protocol.

### Enzyme activity measurements

#### Measurement of γ-secretase activity

Measurement of γ-secretase activity as described earlier (Grimm et al., 2008, 2011b, 2012a, 2014, 2015; Rothhaar et al., 2012; Burg et al., 2013): Briefly, for determination of γ-secretase activity in living cells fluorogenic γ-secretase substrate NMA-GGVVIATVK(DNP)-*D*R*D*R*D*R-NH2 (Calbiochem, Darmstadt, Germany) was used as described in detail earlier (Grimm et al., 2015). Resulting fluorescence was determined continuously at an excitation wavelength of 355 ± 10 nm and an emission wavelength of 440 ± 10 nm for 1 h at 37°C under light exclusion using an Infinite M1000Pro Fluorometer (Tecan, Crailsheim, Germany). The γ-secretase activity in living cells was calculated by subtracting the unspecific turnover determined by adding 2 μM of the highly specific γ-secretase inhibitor X (Shearman et al., 2000). It should be mentioned that similar results showing a decreased γ-secretase activity for several PS1 FAD mutations were observed by us and others in previous studies confirming our assay and the obtained data (Walker et al., 2005; Wiley et al., 2005; Bentahir et al., 2006; Pinnix et al., 2013; Grimm et al., 2014).

For measurement of γ-secretase activity in purified membranes, cell homogenates were adjusted to equal protein amount prior to centrifugation at 900 rcf for 10 min at 4°C. The obtained post-nuclear fractions were centrifuged at 55.000 rpm for 75 min at 4°C. Pelleted purified membranes were resuspended in sucrose buffer and dispensed on black 96-well plates. After addition of 10 μM γ-secretase substrate fluorescence was measured as described above. Unspecificity of this method was between 10 and 30% as published earlier (Rothhaar et al., 2012; Burg et al., 2013).

#### Measurement of NEP activity

After chemical lysis of the cells in lysis buffer (0.5% TritonX-100, 20 mM Tris pH 7.4, 10% Sucrose) NEP activity assay was performed according to Miners et al. (2008b) with minor modifications utilizing the anti-NEP antibody AF1182 (R&D Systems, Minneapolis, Minn., USA) and 5 μM MCA-RPPGFSAFK(DNP)-OH fluorogenic peptide substrate (R&D Systems, Minneapolis, Minn., USA). Fluorescence was measured with an excitation wavelength of 320 ± 10 nm and an emission wavelength of 405 ± 10 nm in a Safire2 Fluorometer (Tecan, Crailsheim, Germany) as described above. Unspecificity of the assay was 22.1% (± 2.4%, *p* ≤ 0.001) as measured in presence of 10 μM thiorphan (Santa Cruz Biotechnology, Dallas, USA) (Supplementary Figure 1A).

#### Measurement of IDE activity

After cells were lysed in lysis buffer (0.5% TritonX-100, 20 mM Tris pH 7.4, 10% Sucrose) IDE activity assay was performed as described in Miners et al. (2008a) with minor modifications utilizing the anti-IDE antibody ST1120 (Calbiochem, Darmstadt, Germany) and 10 μM MCA-RPPGFSAFK(DNP)-OH fluorogenic peptide substrate (R&D Systems, Minneapolis, Minn., USA). Resulting fluorescence was measured at an excitation wavelength of 320 ± 10 nm and an emission wavelength of 405 ± 10 nm in a Safire2 Fluorometer as described above.

#### Measurement of total Aβ degradation

For determination of total Aβ degradation cells were lysed in lysis buffer (150 mM NaCl, 50 mM Tris/HCl pH 7.4, 2 mM EDTA, 0.1% NP-40, 0.1% Triton-X 100). 60 μg total protein of each sample was incubated with 1 μg/ml synthetic human Aβ40 peptide in 100 μl PBS for 1 h at 37°C under gentle shaking. For determination of specific NEP mediated Aβ degradation, samples were incubated additionally with 50 μM thiorphan. Quantification of the remaining, not degraded Aβ was carried out with W02-antibody, only detecting the supplemented human but not the endogenous murine Aβ of the cells. Aβ degrading activity in the lysates was calculated as reciprocal value of the quantified remaining Aβ peptides.

### Statistical analysis

All quantified data represent an average of at least three independent experiments. Error bars represent standard deviation of the mean. Statistical significance was calculated using two-tailed Student's *t*-test; significance was set at ^*^p ≤ 0.05; ^**^p ≤ 0.01 and ^***^p ≤ 0.001.

## Results

### NEP gene expression, protein level and enzyme activity is decreased in absence of PS1/PS2 or APP/APLP2

**Figure 1:**
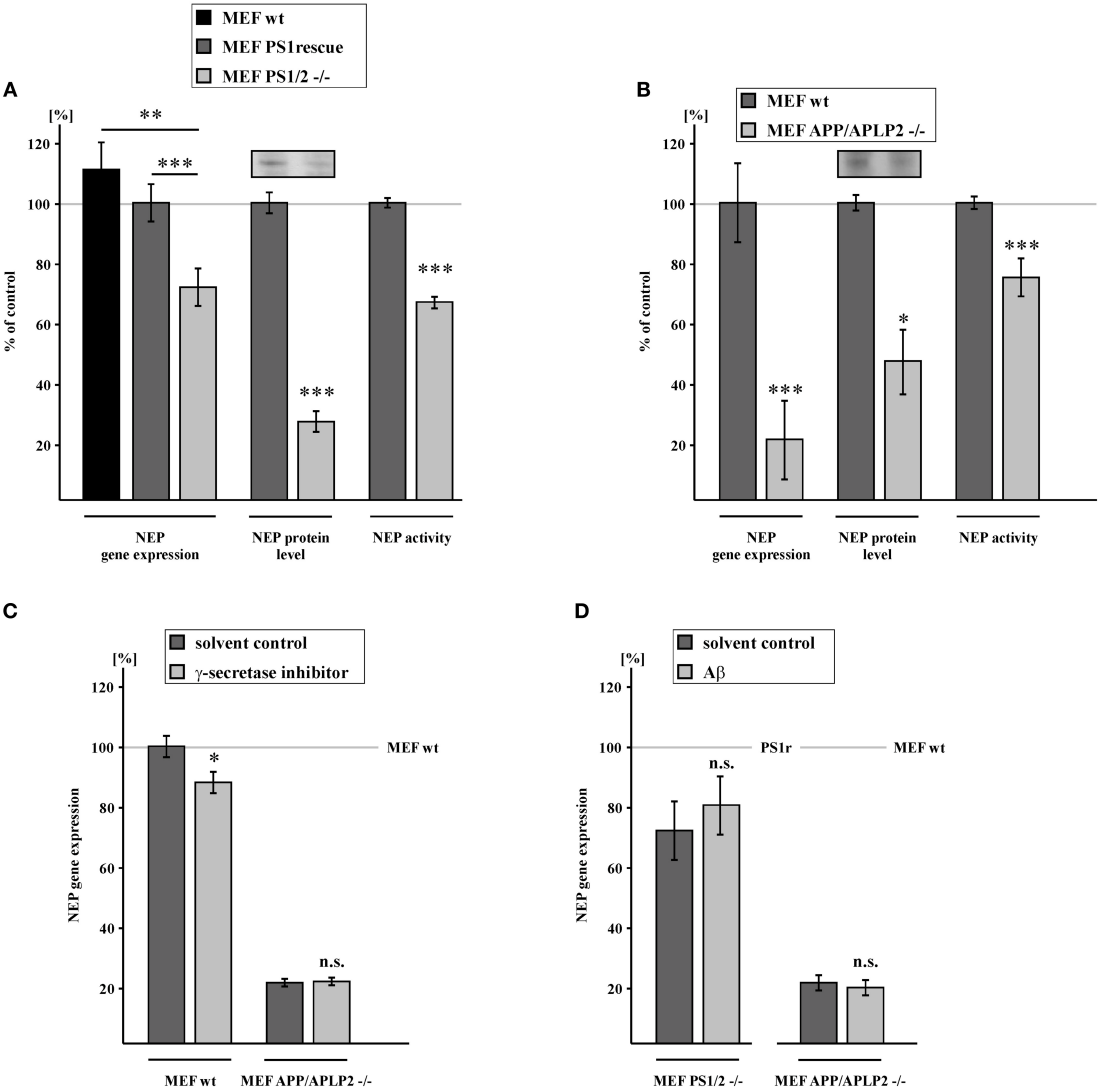
**Reduction of NEP gene expression, protein level and activity in PS1/2 and APP/APLP2 deficient mouse embryonic fibroblasts (MEF). (A)** Reduced NEP gene expression (71.6 ± 6.4%, *p* ≤ 0.001), protein level (26.2 ± 3.5%, *p* ≤ 0.001) and activity (66.6 ± 1.9%, *p* ≤ 0.001) in MEF cells devoid of PS1 and PS2 (MEF PS1/2 ^−/−^) compared to MEF PS1/2 ^−/−^ retransfected with PS1 wt (MEF PS1rescue). Level of NEP gene expression in MEF wt: 111.2 ± 9.2%, *p* = 0.005 when compared to MEF PS1/2 ^−/−^. **(B)** Decreased NEP gene expression (20.4 ± 13.3%, *p* ≤ 0.001), protein level (50.4 ± 10.9%, *p* = 0.012) and activity (74.9 ± 6.4%, *p* ≤ 0.001) in MEF lacking APP and the APP-like protein APLP2 (MEF APP/APLP2 −/−) in comparison to wild-type cells (MEF wt). **(C)** Decreased NEP gene expression in MEF wt treated with γ-secretase inhibitor (87.8 ± 3.6%, *p* = 0.015). Unaltered NEP gene expression in MEF APP/APLP2 −/− after incubation with γ-secretase inhibitor (20.8 ± 1.3%, *p* = 0.73 compared to 20.4% in cells treated with solvent control). **(D)** No effect of Aβ40/42 peptide long term incubation on NEP gene expression in MEF PS1/2^−/−^ (80.2 ± 9.9%, *p* = 0.43 compared to 71.6% in cells treated with solvent control) and MEF APP/APLP2^−/−^ (18.8 ± 2.5%, *p* = 0.43 compared to 20.4% in cells treated with solvent control). Asterisks show the statistical significance (^*^p ≤ 0.05, ^**^p ≤ 0.01 and ^***^p ≤ 0.001, n.s., not significant). All quantified data represent an average of at least three independent experiments. Error bars represent standard deviation of the mean.

To investigate a potential role of the catalytically active part of the γ-secretase complex in the regulation of NEP, we used PS1/PS2-deficient MEFs (MEF PS1/2^−/−^) and PS1 wt retransfected control cells (MEF PS1rescue) to obtain clonal homogeneity. RT-PCR analysis revealed that NEP gene expression was reduced to 71.6% in absence of PS1 and PS2 compared to MEF PS1 rescue. The effect in respect of NEP expression was also confirmed by comparing MEF devoid of PS not only with PS1rescue, but also with wt cells, further emphasizing that the effects are independent of the used control. In accordance with decreased NEP expression, the protein level of NEP as well as NEP activity was also significantly reduced to 26.2 and 66.6% in MEF PS1/2^−/−^ cells vs. MEF PS1rescue (Figure 1A, Supplementary Figure 1B). It has to be pointed out, that effect strength between expression, protein and activity differs. Whereas NEP activity was measured out of total lysates, the protein levels were determined after a membrane protein preparation, which might contribute to the observed differences. Additionally, protein stability or differences in protein translation of NEP might also result in differences in the effect strength. Nevertheless, independent of the used method at every stage NEP is significantly reduced in PS deficient cells, beginning from its expression, to the protein level finally resulting in a decreased enzyme activity. Similar results were obtained in cells devoid of the APP-family. To elucidate whether APP or the APP-like proteins APLP1 or APLP2 or PS-dependent cleavage products of the APP-family are involved in NEP regulation, we used MEF lacking APP and APLP2 (MEF APP/APLP2^−/−^) and thus devoid of the hole APP-family as APLP1 expression is restricted to neurons (Tanzi et al., 1988; Slunt et al., 1994; Lorent et al., 1995). For MEF APP/APLP2^−/−^ cells, RNA level of NEP were even reduced to 20.4% compared to wt cells, indicating that the APP-family or fragments thereof are involved in transcriptional regulation of NEP. As a consequence of decreased NEP gene expression, MEF APP/APLP2^−/−^cells also revealed a reduction in the NEP protein level and NEP enzyme activity to 50.4 and 74.9%, respectively (Figure 1B, Supplementary Figure 1C). Taken into consideration that the PS-dependent APP cleavage product AICD has been suggested to increase NEP gene transcription (Grimm et al., 2013), one might expect a very similar reduction in the NEP RNA level for MEF PS1/2^−/−^ cells and MEF APP/APLP2^−/−^ cells, both cell lines lacking AICD and the intracellular domains of APLP1 and APLP2, ALID 1 and ALID2. However, as γ-secretase activity in MEF PS1rescue cells accounted only for 49.8% (± 7.3%, *p* = 0.004) compared to MEF wt cells (Supplementary Figure 1D), it is not astonishing that the effect on NEP gene expression is less pronounced in MEF PS1/2^−/−^ vs. MEF PS1rescue cells in comparison to the experimental setting comparing MEF APP/APLP2^−/−^ cells with MEF wt cells. It has to be mentioned that the effect strength of AICD on NEP gene expression seems not to be direct proportional to the amount of AICD. Whereas PS1rescue cells have 50% less γ-secretase activity, the NEP expression compared to wt cells NEP gene expression is only slightly decreased. Several reasons, such as clonal heterogeneity, might contribute to this finding. Nevertheless, it cannot be ruled out that e.g. ceiling effects above a certain AICD concentration occur or other additional factors might also influence NEP expression. Although the effect strength on NEP expression is not directly linear to the AICD level, a correlation between NEP expression and AICD amount was observed. To determine whether PS1 indeed regulates NEP through processing of APP, we inhibited γ-secretase activity in MEF APP/APLP2^−/−^ cells and MEF wt cells. Treatment with γ-secretase inhibitor resulted in a significantly decreased NEP gene expression to 87.8% in MEF wt. In contrast, inhibition of γ-secretase activity had no effect on NEP expression in APP/APLP2-deficient cells, clearly demonstrating NEP gene expression to be regulated by a γ-secretase derived APP/APLP2 cleavage product (Figure 1C). To further elucidate the mechanism how PS and the APP-family regulate NEP expression, we incubated APP/APLP2- and PS1/PS2-deficient MEFs with Aβ40/42 peptides over a period of 9 days with intermediate medium changes. Treatment with Aβ40/42 peptides had no significant effect on NEP gene expression in both cell lines indicating NEP expression not to be regulated by Aβ peptides (Figure 1D).

### AICD mediates gene regulation of NEP

**Figure 2:**
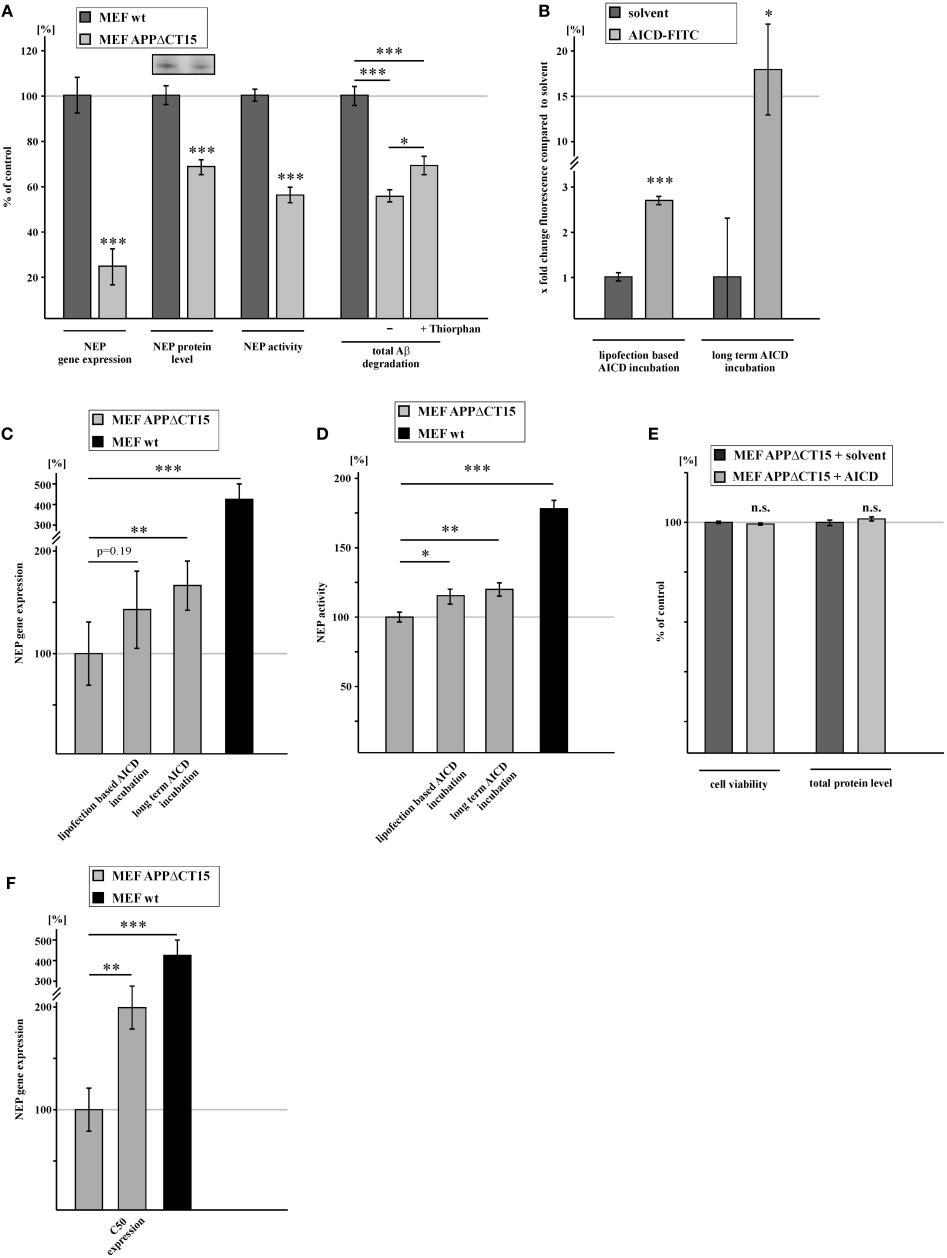
**NEP gene expression and activity is regulated by AICD. (A)** Reduced NEP gene expression (23.4 ± 8.1%, *p* ≤ 0.001), protein level (68.0 ± 4.1%, *p* ≤ 0.001), activity (55.3 ± 3.4%, *p* ≤ 0.001) and total Aβ degradation (−Thiorphan: 54.7 ± 2.7%, *p* ≤ 0.001 compared to wt; +Thiorphan: 68.5 ± 4.1%, *p* ≤ 0.001 compared to wt; *p* = 0.02 for effects −Thiorphan vs. +Thiorphan) in MEF expressing an APP construct lacking the last 15 C-terminal amino acids (aa) and therefore a functional AICD domain (MEF APPΔCT15) compared to control fibroblasts. **(B)** Measurement of FITC-AICD uptake in lysates of MEF APPΔCT15 after short term (2.8 ± 0.1%, *p* ≤ 0.001, shown in x fold change of fluorescence) and long term incubation with FITC-AICD (18.0 ± 5.1%, *p* = 0.03, shown in x fold change of fluorescence) compared to cells treated with solvent control. **(C)** Enhanced NEP gene expression in MEF APPΔCT15 after lipofection based short term (12 h) (143.6 ± 29.8%, *p* = 0.193 compared to solvent control) and after long term (9 days) (168.0 ± 18.7%, *p* = 0.002 compared to solvent control) incubation with AICD peptides. Level of NEP gene expression in MEF wt cells (427.0 ± 74.0%, *p* ≤ 0.001 when compared to MEF APPΔCT15) indicates a partial rescue of NEP gene expression after AICD incubation. **(D)** Increased NEP activity in MEF APPΔCT15 after lipofection based short term (12 h) (115.8 ± 5.5%, *p* = 0.014 compared to solvent control) and after long term (9 days) (120.4 ± 4.9%, *p* = 0.008 compared to solvent control) incubation with AICD peptides. Level of NEP activity in MEF wt cells (180.0 ± 6.12%, *p* ≤ 0.001 when compared to MEF APPΔCT15) indicates a partial rescue of NEP activity after AICD incubation. **(E)** Unaltered viabiliy (99.98 ± 0.11%, *p* = 0.86) and total protein level (101.5 ± 0.87%, *p* = 0.33) in MEF APPΔCT15 incubated with AICD peptides. (**F)** Increase in NEP gene expression (201.9 ± 21.4%, *p* = 0.009) in MEF APPΔCT15 retransfected with the last 50 C-terminal aa of APP (MEF APPΔCT15 + C50) compared to MEF APPΔCT15. Level of NEP gene expression in MEF wt cells (427.0 ± 74.0%, *p* ≤ 0.001 when compared to MEF APPΔCT15) indicates a partial rescue of NEP gene expression due to expression of C50. Statistical significance as described for Figure 1.

To elucidate the impact of AICD on NEP expression, we analyzed APP knock-in MEF devoid of full-length APP, expressing an APP construct lacking the last 15 aa from the C-terminus (MEF APPΔCT15) (Ring et al., 2007). The deleted 15 aa include the NPXY motif of APP, which apparently plays a crucial role in regulating transcription of several genes by interacting with phosphotyrosine-binding (PTB) domain containing adaptor proteins (von Rotz et al., 2004; Uhlik et al., 2005). Therefore, cells expressing the truncated APP construct are lacking a functional AICD domain. Indeed, RNA levels of NEP were strongly reduced to 23.4% in MEF APPΔCT15 cells (Figure 2A). This reduction was nearly identical to the decrease of NEP expression to 20.4% observed for MEF APP/APLP2^−/−^ cells, suggesting that the PS-dependent APP cleavage product AICD mainly regulates NEP gene transcription and that the intracellular domains of APLP1 and APLP2, ALID1 and ALID2, might play no or only a minor role in regulating NEP expression. In addition to reduced gene expression of NEP, MEF APPΔCT15 cells also showed a decreased NEP protein level and enzyme activity to 68.0 and 55.3%, respectively (Figure 2A, Supplementary Figure 1E). Additionally total degradation of human Aβ40 in lysates of MEF APPΔCT15 was decreased to 54.7% in absence and to 68.5% in presence of thiorphan compared to MEF wt, indicating a contribution of altered NEP activity to the effects on total Aβ degradation mediated by a concurrence of several proteases (Figure 2A). To further substantiate the finding that AICD is involved in NEP gene transcription, we incubated MEF APPΔCT15 cells with synthetic AICD peptides, corresponding to the last 20 aa of the APP C-terminus. Incubated AICD peptides were efficiently taken up (Figure 2B) and were shown to be localized in the nuclear compartment earlier (Robinson et al., 2014). Biological activity of incubated AICD peptides has also been demonstrated earlier in several studies (Grimm et al., 2011a, 2012b, 2014; Robinson et al., 2014). For short time incubation, cells were treated for 12 h with 2.5 μM AICD peptide using the protein delivery reagent Saint PhD. NEP gene expression was increased to 143.6% in AICD treated MEF APPΔCT15 cells compared to untreated cells lacking functional AICD, however the obtained increase was not statistically significant. Nevertheless, long term incubation with 2 μM AICD for 9 days with intermediate medium changes to avoid AICD degradation revealed a statistically significant increase of NEP expression to 168.0% in AICD treated MEF APPΔCT15 cells (Figure 2C), illustrating the necessity to combine different experimental settings to clarify the role of AICD in gene transcription. The increase in NEP gene expression after AICD incubation corresponds to a partial rescue of 13.3% for short term and of 20.8% for long term AICD treatment compared to NEP expression level in MEF wt cells. Enzyme activity of NEP was also significantly elevated in AICD treated cells, for both short and long term AICD incubation (MEF APPΔCT15 + AICD 12 h, activity: 115.8% corresponding to 19.8% rescue; + AICD 9d, activity: 120.4% corresponding to 25.5% rescue (Figure 2D, Supplementary Figures 1F,G). To exclude a toxic effect of truncated AICD peptides (Passer et al., 2000), we determined total protein level and cell viability in MEF APPΔCT15 cells after short term incubation with truncated AICD peptides. Neither total protein level nor cell viability measured by LDH release was altered in AICD treated cells in comparison to cells treated with solvent control (Figure 2E). The function of AICD in regulating NEP expression was further validated by analyzing MEF APPΔCT15 cells transiently retransfected with C50, representing AICD generated by ε-cleavage of APP (Gu et al., 2001; Yu et al., 2001). C50 expression resulted in a significant increase of NEP expression to 201.9% corresponding to a partial rescue of 31.2% compared to NEP gene expression level in MEF wt cells (Figure 2F). When IDE was additionally transiently knocked down to 70.8% (± 3.8%, *p* = 0.0016) in C50 expressing cells, NEP expression was slightly increased to 108.7% (± 2.9%, *p* = 0.038) compared to C50 cells.

### β-secretase mediated APP cleavage generates transcriptionally active AICD

**Figure 3:**
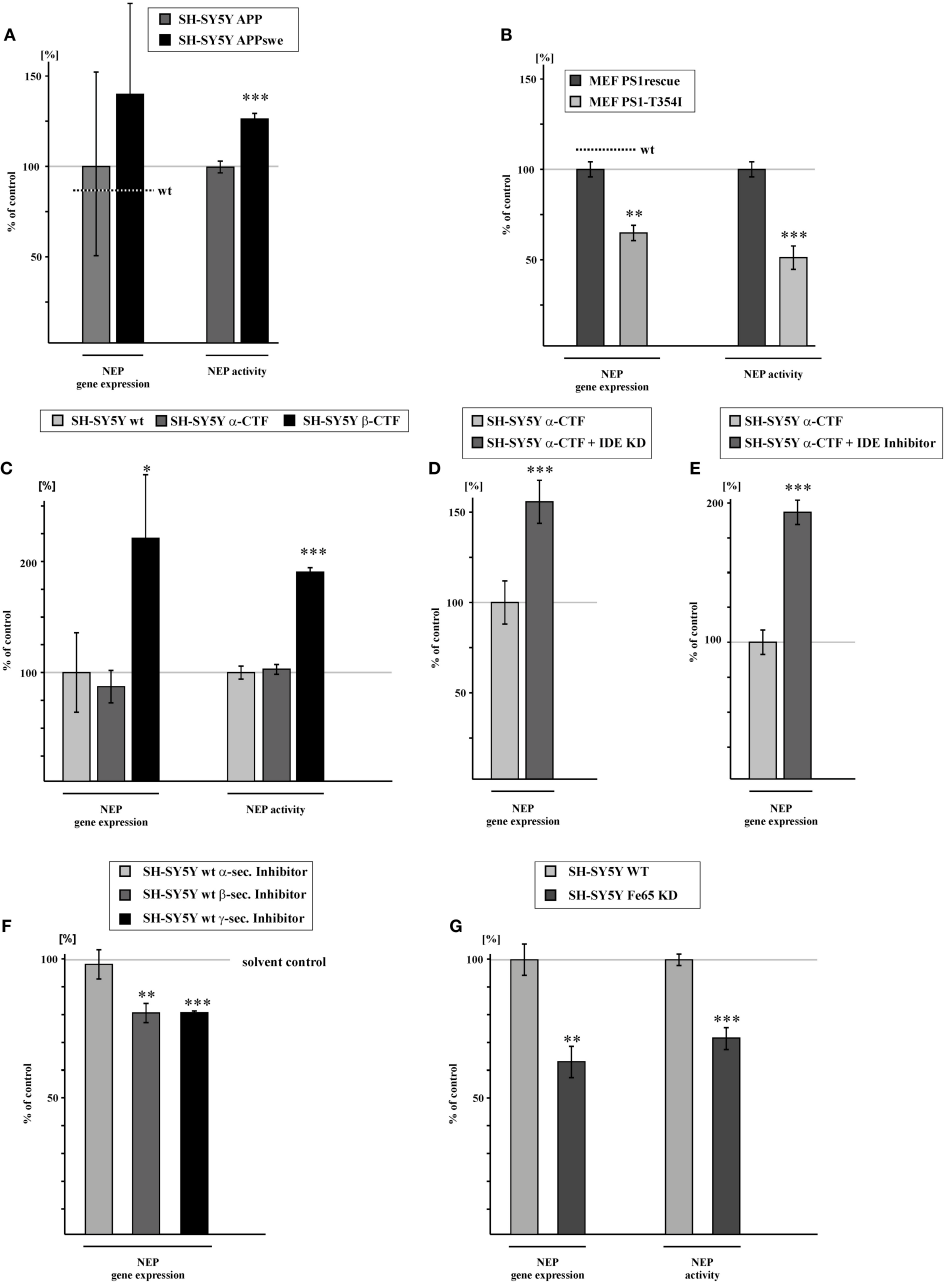
**NEP gene expression and activity is influenced by amyloidogenic, but not by non-amyloidogenic APP processing due to degradation of α-/γ-APP-cleavage derived AICD by IDE. (A)** Increased NEP gene expression (139.6 ± 52.3%, *p* = 0.49) and activity (127.5 ± 3.3%, *p* ≤ 0.001) in SH-SY5Y cells overexpressing APP carrying the swedish mutation (APPswe) compared to SH-SY5Y APP cells. Level of NEP gene expression in SH-SY5Y wt cells is indicated by a dotted line (p = 0.01 for NEP gene expression in SH-SY5Y APPswe compared to SH-SY5Ywt). **(B)** Reduction of NEP gene expression (64.0 ± 4.4%, *p* = 0.0012) and activity (50.0 ± 5.6%, *p* ≤ 0.001) in MEF PS1/2 deficient cells retransfected with PS1 carrying the T354I mutation (MEF PS1-T354I) compared to MEF PS1/2 deficient cells retransfected with PS1 wt (MEF PS1rescue). Level of NEP gene expression in MEF wt cells is indicated by a dotted line. **(C)** NEP gene expression and activity in SH-SY5Y overexpressing the APP α-cleaved C-terminal fragment (α-CFT) (RNA-level: 86.9 ± 9.5%, *p* = 0.241; activity: 100.3 ± 6.0%, *p* = 0.967) and the APP β-cleaved C-terminal fragment (β-CFT) (RNA-level: 223.5 ± 36.3%, *p* = 0.027; activity: 192.5 ± 3.9%, *p* ≤ 0.001). **(D)** Increased NEP gene expression (157.2 ± 12.2%, *p* ≤ 0.001) in SH-SY5Y α-CFT cells with reduced IDE expression (SH-SY5Y α-CTF + IDE-KD). **(E)** Enhanced NEP expression (195.2 ± 6.9%, *p* ≤ 0.001) in SH-SY5Y α-CTF cells after pharmacological IDE inhibition (SH-SY5Y α-CTF + IDE inhibitor). **(F)** NEP gene expression in SH-SY5Y wt cells treated with α- (98.3 ± 5.4%, *p* = 0.764), β - (80.6 ± 3.5%, *p* = 0.005) or γ- (80.7 ± 0.6%, *p* ≤ 0.001) secretase inhibitor. **(G)** Reduction in NEP gene expression (63.0 ± 5.1%, *p* = 0.002) and activity (71.5 ± 4.0%, *p* ≤ 0.001) in SH-SY5Y Fe65 knock-down (SH-SY5Y Fe65-KD) compared to mock-transfected control cells. Statistical significance as described for Figure 1.

Familial mutations in the genes encoding APP or PS1 and PS2 are frequently used to describe the pathological situation of AD, but they can also be beneficial for mechanistic studies. The familial Swedish mutation of APP (APPswe) (Mullan et al., 1992), a double mutation at the β-secretase cleavage site, results in a strong increase of total Aβ level, as APPswe is preferentially processed by the amyloidogenic β-secretase pathway instead of non-amyloidogenic α-secretase processing (Citron et al., 1992; Felsenstein et al., 1994). As β-secretase mediated APP cleavage in endosomal compartments is considered mainly responsible for the generation of transcriptionally active AICD species (Belyaev et al., 2010; Flammang et al., 2012), one might expect increased NEP gene transcription in APPswe expressing cells. Indeed, human neuroblastoma SH-SY5Y cells, stably transfected with APPswe showed a strong but not statistically significant increase in NEP expression to 139.6% compared to SH-SY5Y cells stably expressing APPwt. The effect on NEP gene expression is statistically significant when comparing SH-SY5Y APPswe to SH-SY5Y wt cells. Along with elevated NEP expression, NEP enzyme activity was also increased to 127.5% in APPswe cells (Figure 3A, Supplementary Figure 1H). In contrast to the APPswe mutation affecting β-secretase cleavage, mutations in the catalytically active presenilins affect γ-secretase processing of APP, mainly by shifting the Aβ42/40 ratio towards the generation of the more hydrophobic Aβ42 species (Duff et al., 1996; Citron et al., 1997; Duering et al., 2005). However, several studies have revealed that some of the known PS mutations also reduce γ-secretase processing of APP resulting in decreased AICD levels compared to cells expressing wt presenilins (Walker et al., 2005; Wiley et al., 2005; Bentahir et al., 2006; Pinnix et al., 2013). As a genetic approach we analyzed PS-deficient MEFs retransfected with the FAD PS1 mutation T354I (MEF PS1-T354I) compared to PS1 wt (MEF PS1rescue). This mutation is known to decrease γ-secretase activity (Grimm et al., 2014). RT-PCR analysis showed that NEP expression is reduced to 64% in MEF PS1-T354I expressing cells compared to MEF PS1rescue. NEP activity was also decreased to 50.0% in these cells (Figure 3B, Supplementary Figure 1I). Further characterization of the PS1 mutation revealed that γ-secretase activity in living cells is indeed reduced to 75.2% (± 2.4%, *p* ≤ 0.001) in MEF PS1-T354I (Supplementary Figure 1J) suggesting that the coincidental reduction in AICD generation is responsible for the decrease in NEP expression.

To further clarify the involvement of β - and α-secretase mediated APP processing in the generation of transcriptionally active AICD peptides, we stably transfected SH-SY5Y cells with α-CTF or β-CTF. Notably, NEP gene expression was not significantly altered for α-CTF expressing SH-SY5Y cells, whereas gene transcription of NEP was significantly elevated to 223.5% in β-CTF expressing cells compared to SH-SY5Y wt cells. Similar results were found for NEP activity. Enzyme activity of NEP was unaltered for α-CTF expressing cells, but strongly increased to 192.5% for β-CTF expressing cells (Figure 3C, Supplementary Figure 1K). However, when SH-SY5Y IDE knock-down (KD) cells (IDE RNA level: 75.9 ± 10.1%, *p* = 0.029) were transiently transfected with α-CTF, we observed an increase in NEP gene expression to 157.2% (Figure 3D). Additionally, we inhibited IDE by incubating SH-SY5Y cells stably expressing α-CTF with 10 μM insulin, which results in inhibition of IDE activity to 63.1% (± 2.12%, *p* = 0.0018) (Supplementary Figure 1L). This resulted in a significant increase in NEP expression to 195.2% (Figure 3E). These findings indicate that AICD generated by α-secretase processing is rapidly degraded by IDE (Edbauer et al., 2002; Farris et al., 2003) and thus not transcriptionally active, whereas β-secretase generated AICD seems to be more stable, possibly by rapid binding to adaptor proteins like Fe65, known to stabilize AICD (Kimberly et al., 2001; Kinoshita et al., 2002). If the hypothesis is that α-secretase generated AICD is not transcriptionally active, one would expect that inhibition of α-secretase activity has no effect on NEP gene transcription. In fact, treatment of SH-SY5Y wt cells with an α-secretase inhibitor showed unaltered NEP expression, whereas in the presence of a β-secretase inhibitor RNA level of NEP were decreased to 80.6% (Figure 3F). As a control for α- and β-secretase inhibition, we analyzed sAPPα and sAPPβ level in the cell culture media of inhibitor-incubated cells. As expected sAPPα as well as sAPPβ secretion were strongly reduced in presence of the inhibitors (sAPPα: 38.1 ± 4.5%, *p* ≤ 0.001; sAPPβ : 6.3 ± 0.7%, *p* ≤ 0.001). Treatment of SH-SY5Y wt cells with a γ-secretase inhibitor also resulted in a reduction of NEP expression to 80.7% (Figure 3F), identical to the reduction observed for β-secretase inhibition. Efficiency of γ-secretase inhibitor treatment was verified by analyzing total Aβ level in the medium of inhibitor-treated cells which were reduced to 52.2% (± 7.5%, *p* = 0.004) compared to cells treated with solvent control. To further validate a function of AICD in regulating gene expression of NEP, we knocked-down Fe65, one of the adaptor proteins known to bind to the APP C-terminus and suggested to stabilize AICD peptides (Kimberly et al., 2001; Kinoshita et al., 2002; Cao and Sudhof, 2004). Fe65 knocked-down SH-SY5Y wt cells showed a 59% reduction of Fe65 expression (Fe65 RNA level: 41.2 ± 1.9%, *p* ≤ 0.001) compared to SH-SY5Y wt cells. Notably, Fe65 KD cells also revealed a strong reduction of NEP expression to 63.0% and a decrease in NEP activity to 71.5% (Figure 3G, Supplementary Figure 1M), indicating that Fe65 is important for AICD-mediated gene regulation of NEP.

### Gene expression of NEP is also affected *in vivo* in different transgenic mouse models

**Figure 4:**
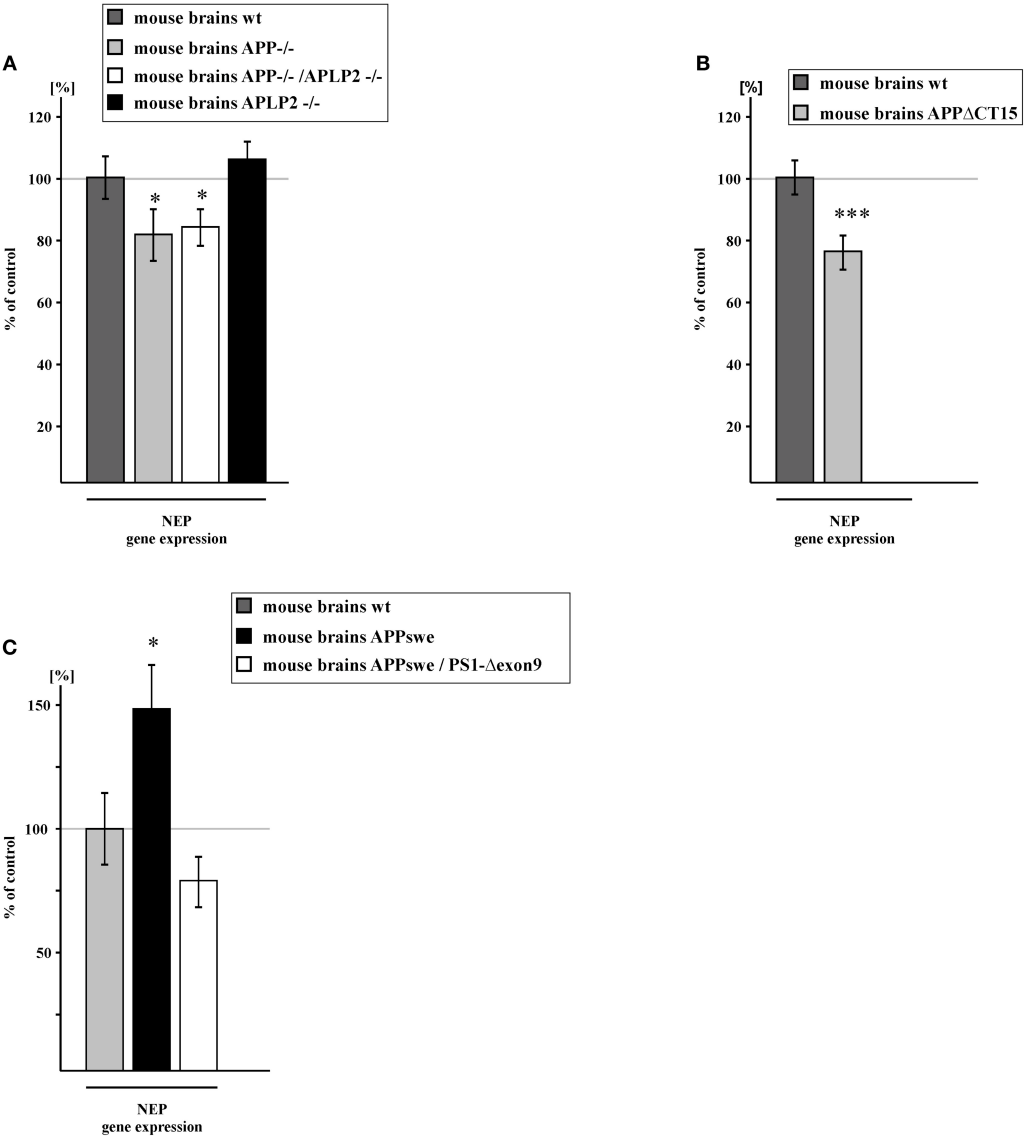
**NEP gene expression in brain tissue of transgenic mice. (A)** NEP gene expression in brain tissue of APP knock-out (APP^−/−^) (81.3 ± 8.5%, *p* = 0.049), APLP2 knock-out (APLP2^−/−^) (106.2 ± 6.1%, *p* = 0.317) and in APP/APLP2-double knockout mice (APP/APLP2^−/−^) (83.8 ± 6.1%, *p* = 0.015). **(B)** NEP gene expression in mice devoid of the last 15 APP C-terminal aa and hence AICD (APPΔCT15) (75.8 ± 5.7%, *p* ≤ 0.001). **(C)** NEP gene expression in APPswe (149.5 ± 19.4%, *p* = 0.016) and APPswe/PS1ΔE9 (78.6 ± 10.5%, *p* = 0.058) mouse brains. Statistical significance as described for Figure 1.

Similar results regarding the regulation of NEP expression were obtained *in vivo* by analyzing transgenic AD mouse brains. NEP expression was significantly reduced to 81.3% in APP knock-out (APP^−/−^) mouse brains compared to wt mouse brains. Importantly, APP/APLP2 knock-out (APP/APLP2^−/−^) mouse brains showed a reduction of NEP expression to 83.8% and thus nearly identical to the reduction obtained for APP^−/−^ mouse brains. This indicates that the intracellular domain of APP, AICD, but not the intracellular domain of APLP2, ALID2, is involved in the regulation of NEP. This finding could be substantiated by analyzing APLP2 single knock-out (APLP2^−/−^) mouse brains, which revealed no significant changes in NEP gene expression (Figure 4A). The importance of AICD in regulating NEP *in vivo* could be further validated by the use of mouse brains expressing the truncated APP construct APPΔCT15, revealing a decrease of NEP expression to 75.8% (Figure 4B). In line with our cell culture findings, brains of APPswe transgenic mice revealed an elevation of NEP expression to 149.5% compared to wt mouse brains. In contrast, NEP expression was reduced to 78.6% in brains of APPswe/PS1-Δexon9 double transgenic mice, in which γ-secretase processing and thus AICD generation is reduced (Bentahir et al., 2006) (Figure 4C).

## Discussion

Since identification of striking similarities between the proteolytic processing of Notch and APP there has been controversy on whether AICD mediates gene transcription in analogy to the Notch intracellular domain NICD. Although several AICD-regulated target genes have been identified (Pardossi-Piquard and Checler, 2012; Grimm et al., 2013), a putative role of AICD in gene transcription of NEP, one of the most important Aβ-degrading enzymes, is still disputable. The inconsistent results addressing AICD-mediated NEP regulation and other target genes in general might be caused by rapid degradation of AICD by IDE and other proteases once it is released to the cytosol (Cupers et al., 2001; Edbauer et al., 2002; Farris et al., 2003), complicating the finding of experimental conditions to identify the physiological function of AICD. Further experimental details, e.g., analyzed cell types or tissues, knock-out vs. overexpressing cell culture systems, single vs. double transgenic mouse models etc. might further contribute to the conflicting outcomes of different studies (Pardossi-Piquard et al., 2007; Bauer et al., 2011). Therefore, the present study analyzed diverse knock-out systems *in vitro* and *in vivo* as well as several overexpressing cell culture systems to further clarify the involvement of PS-dependent γ-secretase processing of the APP-family in NEP expression and to identify whether amyloidogenic β-secretase or non-amyloidogenic α-secretase processing generates transcriptionally active AICD peptides. Furthermore, we used AICD peptide supplementation or pharmacological inhibition of AICD generation.

Consistent with recent findings (Pardossi-Piquard et al., 2005; Chen and Selkoe, 2007) we found that NEP expression as well as NEP protein level and activity were strongly reduced in MEF devoid of PS1/PS2 or APP/APLP2, indicating that γ-secretase dependent cleavage products of the APP-family are involved in gene regulation of NEP. In contrast, one study (Huysseune et al., 2009) reported unaltered gene expression level of NEP for PS1/2-deficient MEF and unchanged NEP protein level for MEF devoid of APP and APLP2. In support of this, another study (Hébert et al., 2006) found no alterations in the NEP protein level in PS1/2-deficient and APP/APLP2-deficient cells, illustrating the problematic of divergent results even for identical cell lines and the necessity to combine a variety of adequate experimental settings to clarify a putative role of AICD or ALID1 and ALID2 in gene transcription. Using a truncated APP construct, lacking a functional AICD domain by deletion of the last 15 aa including the NPXY motif (Chen et al., 1990) known to bind to PTB-domain containing adaptor proteins (Cao and Sudhof, 2001; von Rotz et al., 2004; Uhlik et al., 2005; Radzimanowski et al., 2008), we could further validate a physiological role of AICD in transcription. NEP transcription, protein level and enzymatic activity were dramatically reduced in APPΔCT15 cells. Interestingly, fibroblasts lacking a functional AICD domain or APP/APLP2 revealed a very similar reduction in NEP gene expression to 23.4% or 20.45%, respectively, compared to wt fibroblasts. As APP/APLP2-deficient MEFs are also lacking APLP1 (Herms et al., 2004), one might conclude that mainly AICD, but to a lesser extent the γ-secretase-derived fragments of APLP1 (ALID1) and APLP2 (ALID2) are involved in NEP gene transcription. These findings could be confirmed *in vivo* by analyzing APP and APLP2 single or double knock-out mice. Brains of APP^−/−^ and APP/APLP2^−/−^ mice also revealed a nearly identical reduction in NEP transcription to 81.3 and 83.3%, respectively, whereas APLP2 single knock-out mice showed no significant effect on NEP gene expression, indicating that AICD also controls NEP transcription *in vivo*. The *in vivo* function of AICD in the regulation of NEP is confirmed by a study (Pardossi-Piquard et al., 2005) showing reduced NEP activity in APP- and APP/APLP2-deficient mouse brains. Although NEP activity was also reduced to a very similar extent in APP- and APP/APLP2-deficient mouse brains in this study, also indicating that mainly AICD controls cerebral NEP transcription and activity *in vivo*, one cannot exclude that ALID1 and ALID2 might also be involved in the regulation of NEP. Fibroblasts lacking APLP2 revealed reduced NEP expression and enzymatic activity, and NEP activity could be restored by APLP2 retransfection (Pardossi-Piquard et al., 2005). Furthermore, ALID1 and ALID2 have been shown to increase NEP activity in fibroblasts (Pardossi-Piquard et al., 2005). However, similar to our study addressing NEP transcription in APLP2-deficient mice, Chen and Selkoe (2007) reported unchanged NEP protein level and NEP activity for APLP2-deficient mouse brains *in vivo*. In this context it should be mentioned that in contrast to our findings and the findings by Pardossi-Piquard et al. (2005), the authors also did not find alterations in the NEP protein level and NEP activity for APP-deficient mouse brains, supported by unchanged NEP expression level in APP/APLP2-deficient embryonic mouse brains (Hébert et al., 2006). Despite the conflicting data addressing the involvement of γ-secretase dependent cleavage products of the APP-family in the regulation of NEP, we could further demonstrate the importance of AICD in NEP transcription *in vivo* by analyzing APPΔCT15 transgenic mice. The lack of a functional AICD domain resulted in a significant reduction of NEP transcription to 75.8%.

Additionally, in the present study AICD-mediated NEP transcription was further validated by incubating APPΔCT15 cells with AICD peptides, corresponding to the last 20 aa from the APP C-terminus. An exposure of MEF APPΔCT15 cells with AICD peptides for 12 h revealed increased but statistically non-significant gene expression of NEP to 144%, whereas a longer exposure for 9 days resulted in a significant increase in NEP expression to 168%. NEP activity was also increased by both short and long time AICD exposure. To exclude that these changes are caused by the unphysiological nature of the applied AICD peptides, we transiently transfected APPΔCT15 cells with C50, representing physiological AICD peptides generated by γ-secretase dependent ε-cleavage of APP (Gu et al., 2001; Yu et al., 2001). AICD-C50 expression also resulted in a strong increase in NEP gene expression to 202%, further emphasizing that AICD plays a crucial role in transcription by increasing NEP expression. Increased NEP activity and NEP expression was also found for PS-deficient fibroblasts and PS-deficient blastocyst-derived cells (BD8) transiently transfected with AICD-C50 or AICD-C59 (Pardossi-Piquard et al., 2005). In contrast, transient transfection of PS-deficient BD8-cells with AICD-C60 along with the adaptor protein Fe65 and the acetyltransferase Tip60 failed to cause a significant increase in NEP protein level (Chen and Selkoe, 2007). Transcriptome analysis of human neuroblastoma cells inducible for AICD and/or Fe65 expression also failed to identify differential expression of AICD target genes, including NEP (Muller et al., 2007). However, *in vivo* AICD/Fe65 double-transgenic mice displayed enhanced cerebral expression of NEP compared to Fe65 single-transgenic mice (Pardossi-Piquard et al., 2007), substantiating the conclusion that NEP can be transcriptionally controlled by AICD.

The analysis of AICD-mediated gene transcription is further complicated by experimental indications that not all pools of AICD are transcriptionally active. It has been suggested that β-secretase-mediated APP cleavage in endosomal compartments generates AICD peptides active in nuclear signaling (Passer et al., 2000; Goodger et al., 2009). As a cellular model for increased β-secretase processing of APP we used APPswe overexpressing human neuroblastoma cells. SH-SY5Y cells stably expressing the Swedish double mutation showed an increase in NEP transcription to 139.6% and NEP activity to 127.5% compared to SH-SY5Y cells stably expressing APPwt and mainly processed by α-secretase, indicating that amyloidogenic APP processing by β-secretase is involved in the AICD-mediated transcriptional control of NEP. *In vivo*, APPswe transgenic mice also revealed a strong increase in NEP expression to 149.5%. These data are in line with a study (Belyaev et al., 2010) demonstrating that the Swedish double mutation increases NEP expression in cell culture experiments. To exclude that the overexpression of APPswe resulted in a false-positive result, we inhibited β-secretase processing of APP in human neuroblastoma wt cells. As a consequence of reduced β-secretase processing of APP verified by decreased sAPPβ levels in the cell culture medium, NEP transcription was reduced to 80.6%. In contrast, α-secretase inhibition did not cause alterations in NEP gene expression, further indicating that β-secretase processing generates transcriptionally active AICD peptides whereas α-secretase processing of APP seems to be not involved in the generation of AICD peptides active in nuclear signaling. This finding is substantiated by the fact that γ-secretase inhibition, which resulted in divergent outcomes in respect to the regulation of NEP in literature (Pardossi-Piquard et al., 2005; Hébert et al., 2006; Chen and Selkoe, 2007; Xu et al., 2011), also leads to a reduction of NEP expression to 80.7%. The importance of β-secretase processed APP in transcriptional regulation of NEP is further validated by the results obtained by stably expressing the β-secretase cleaved APP fragment β-CTF or the α-secretase cleaved APP fragment α-CTF. Overexpression of β-CTF in SH-SY5Y wt cells resulted in a strong increase in NEP expression to 223.5% and enzymatic activity to 192.5% compared to SH-SY5Y wt cells, whereas overexpression of α-CTF revealed no significant changes, neither in NEP expression nor NEP activity. However, when IDE knock-down cells were transiently transfected with α-CTF, NEP expression was significantly increased to 157%. A strong elevation in NEP expression to 195% was also observed when IDE activity was inhibited by using insulin as an inhibitor in α-CTF expressing cells, demonstrating that IDE is responsible for the degradation of α-secretase generated AICD peptides. Of note, the adaptor protein Fe65 is thought to increase the stability of AICD (Kimberly et al., 2001; Kinoshita et al., 2002), protecting AICD peptides from its degradation by IDE or other proteases (Edbauer et al., 2002; Farris et al., 2003; Nunan et al., 2003; Vingtdeux et al., 2007; Asai et al., 2011). Indeed knock-down of Fe65 in SH-SY5Y wt cells resulted in a significant reduction of NEP expression and NEP activity to 63.0 and 71.5%, respectively. These data support the model proposed by Belyaev et al. (2010), in which AICD peptides generated by α- and γ-secretase processing at the plasma membrane are rapidly degraded by IDE and thus not transcriptionally active, whereas AICD peptides generated by β-secretase processing in the endosomal compartments are active in nuclear signaling because of their rapid binding to Fe65 and subsequent transport to the nucleus, controlling gene transcription of NEP.

In summary, our data show that AICD is able to control NEP expression *in vitro* and *in vivo* and that mainly amyloidogenic APP processing generates transcriptionally active AICD peptides.

## Author contributions

JM, CS, SG, VH, TB, BH, VZ, and NM performed the experiments and analyzed data. UM and HT provided cell lines and murine brain material. MG, HG, JM, and TH prepared the manuscript. MG designed the experiments.

### Conflict of interest statement

The authors declare that the research was conducted in the absence of any commercial or financial relationships that could be construed as a potential conflict of interest.

## Supplementary material

Supplementary Figure 1 **Kinetics of enzyme activities. (A)** NEP activity in MEF wt cells in absence or presence of 10 μM thiorphan. **(B)** NEP activity in MEF PS1/2 −/− and MEF PS1rescue. **(C)** NEP activity in MEF APP/APLP2−/− and MEF wt. **(D)** γ-secretase activity in MEF PS1rescue cells and MEF wt. **(E)** NEP activity in MEF APPΔCT15 and MEF wt. **(F)** NEP activity in MEF APPΔCT15 after lipofection based short term (12 h) incubation with AICD peptides or solvent control. **(G)** NEP activity in MEF APPΔCT15 after long term (9 days) incubation with AICD peptides or solvent control. **(H)** NEP activity in SH-SY5Y APPswe and SH-SY5Y APP. **(I)** NEP activity in MEF PS1-T354I and MEF PS1rescue. **(J)** γ-secretase activity in MEF PS1-T354I and MEF PS1rescue. **(K)** NEP activity in SH-SY5Y α-CTF, SH-SY5Y β-CTF and SH-SY5Y wt. **(L)** IDE activity in SH-SY5Y wt cells incubated with 10 μM insulin or solvent control. **(M)** NEP activity in SH-SY5Y Fe65-KD and control cells.
