# Supplementary figures and images for: APP intracellular domain derived from amyloidogenic β- and γ-secretase cleavage regulates neprilysin expression
Source: Front Aging Neurosci. 2015 May 19;7:77. doi: 10.3389/fnagi.2015.00077 (PMC4443740; doi:10.3389/fnagi.2015.00077)

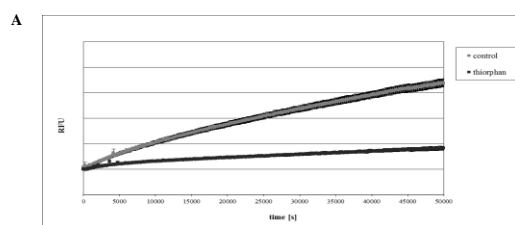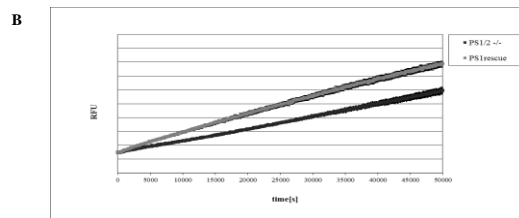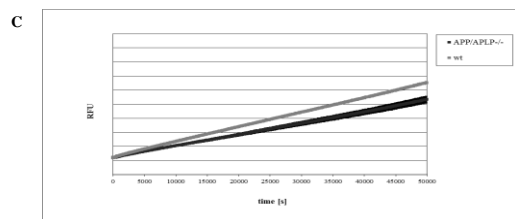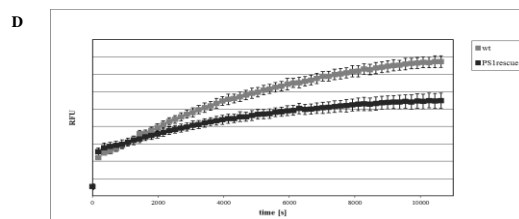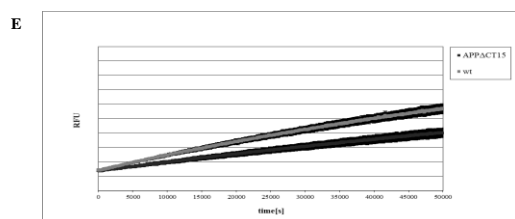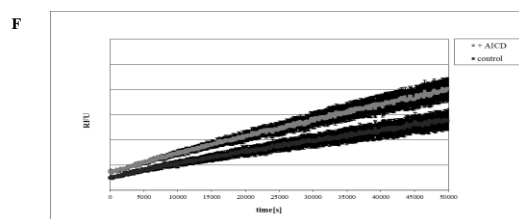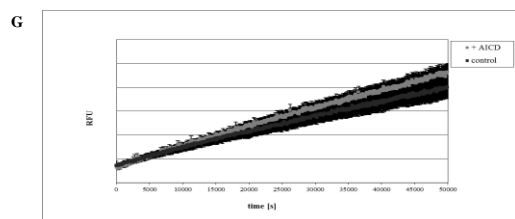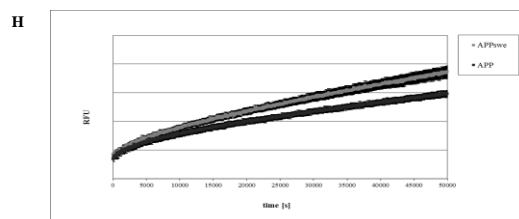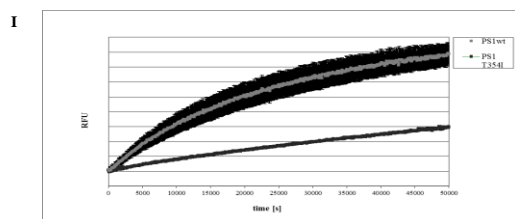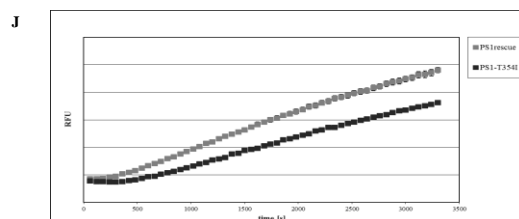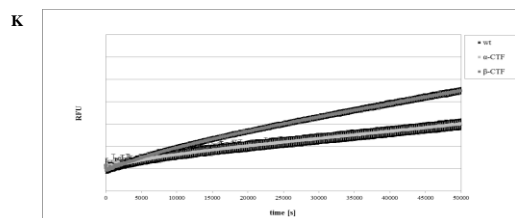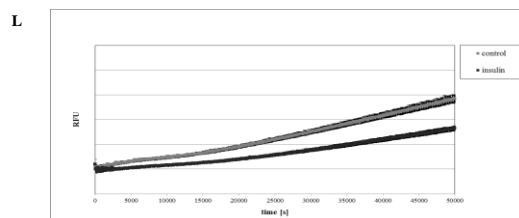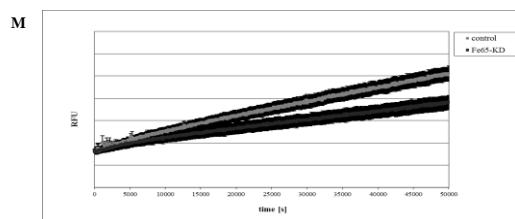

Supplement: Supplementary Figure 1 — Kinetics of enzyme activities. (A) NEP activity in MEF wt cells in absence or presence of 10 μM thiorphan. (B) NEP activity in MEF PS1/2 −/− and MEF PS1rescue. (C) NEP activity in MEF APP/APLP2−/− and MEF wt. (D) γ-secretase activity in MEF PS1rescue cells and MEF wt. (E) NEP activity in MEF APPΔCT15 and MEF wt. (F) NEP activity in MEF APPΔCT15 after lipofection based short term (12 h) incubation with AICD peptides or solvent control. (G) NEP activity in MEF APPΔCT15 after long term (9 days) incubation with AICD peptides or solvent control. (H) NEP activity in SH-SY5Y APPswe and SH-SY5Y APP. (I) NEP activity in MEF PS1-T354I and MEF PS1rescue. (J) γ-secretase activity in MEF PS1-T354I and MEF PS1rescue. (K) NEP activity in SH-SY5Y α-CTF, SH-SY5Y β-CTF and SH-SY5Y wt. (L) IDE activity in SH-SY5Y wt cells incubated with 10 μM insulin or solvent control. (M) NEP activity in SH-SY5Y Fe65-KD and control cells. [file Image1.PDF]
